# Kamikihito Enhances Cognitive Functions and Reward-Related Behaviors of Aged C57BL/6J Mice in an Automated Behavioral Assay System

**DOI:** 10.3389/fphar.2020.01037

**Published:** 2020-07-17

**Authors:** Hiroaki Oizumi, Shinji Miyazaki, Masahiro Tabuchi, Toshihiro Endo, Yuji Omiya, Kazushige Mizoguchi

**Affiliations:** ^1^ Tsumura Kampo Research Laboratories, Tsumura & Co., Ibaraki, Japan; ^2^ Phenovance Research & Technology, LLC, Chiba, Japan

**Keywords:** aging, anxiety, cognitive function, depression, frailty, IntelliCage, kamikihito, kampo (traditional Japanese medicine)

## Abstract

The cognitive and psychological domains of frailty in the elderly have drawn increasing attention given the aging of society. However, therapeutics to treat minor deficits in cognition and mental state in the elderly remain an unmet need. Kamikihito (KKT), a traditional Japanese Kampo medicine indicated for neuroses, anxiety, and insomnia, is effective for treating cognitive dysfunction and depressive-like behaviors in animal models, suggesting that it may have therapeutic potential for treating cognitive and/or mental frailty. In this study, we first validated the known anxiolytic effects of KKT in a conventional maze test. We then introduced an automated behavioral assay system, IntelliCage, to evaluate the therapeutic potential of KKT for age-related and diverse central functions by performing sequential behavioral tasks in young and aged mice to assess basal activities, cognitive functions, perseveration, and hedonic-related behaviors. Although young mice treated with KKT did not exhibit changes in diurnal variation, KKT-administered aged mice exhibited an accelerated decline in voluntary activity during the early part of the light period, implying that KKT may promote sleep onset in aged mice. Neither place learning acquisition for gaining rewards nor subsequent behavioral flexibility performance was altered by KKT in the young group, whereas the aged KKT group exhibited significantly enhanced performance in both phases of learning relative to age-matched controls. Conversely, perseverative nose-pokes (NPs) to gain rewards observed during place learning, indicative of compulsivity, were attenuated by KKT in both age groups. Regarding hedonic processing, aged mice exhibited a decreased preference for sweet solutions compared to young mice, which was effectively reversed by KKT treatment. Furthermore, KKT elevated high-effort choices for high-value reward in an effort-based decision-making paradigm in both age groups, implying augmentation of motivational behaviors by KKT. Collectively, KKT exerted various beneficial effects in cognitive and emotional domains, several of which were more evident in aged mice than in young mice, suggesting the potential of KKT for treating cognitive and mental frailty.

## Introduction

Global population aging has made extending healthy life years a crucial issue. Recent clinical studies have revealed that frailty, a reversible state of accelerated vulnerability to stressors due to age-related and multidimensional declines in physiological systems in elderly people, leads to irreversible health problems and shortened health expectancies (e.g., increase in care home admission, hospitalization, and death) (Fried et al., 2001; Rockwood et al., 2005; Clegg et al., 2013). Physical health domains such as reduced grip strength, slow gait speed, and body weight loss are primarily used to assess frailty in the elderly (Fried et al., 2001). However, increasing focus has been placed on the cognitive and psychological domains of frailty (primarily characterized by subjective cognitive decline or mild cognitive impairment and depressive mood, respectively), as they are associated with increased risk of functional disability, reduced quality of life, incidence of dementia, and mortality, relative to that in elderly people without cognitive or mental abnormalities (Khezrian et al., 2017; Panza et al., 2018; Sugimoto et al., 2018; Shimada et al., 2019). As such, subtle cognitive and psychological dysfunction in the elderly should be pre-emptively addressed to prevent these symptoms from becoming irreversible disability states.

Kamikihito (KKT), a traditional Japanese Kampo medicine, is a crude extract of 14 medicinal herbs originally described in a traditional Chinese medicine textbook “Nei-Ke-Zhai-Yao” published in the sixteenth century. KKT has been approved by the Japanese Ministry of Health, Labour and Welfare since 1986, with indications for anxiety, insomnia, anemia, and neuroses (primarily classified into anxiety, depressive, obsessive–compulsive, and related disorders, as well as somatic symptom and related disorders in the Diagnostic and Statistical Manual of Mental Disorders, Fifth Edition [DSM-5]) in patients with weak constitutions. Although clinical evidence of the efficacy of KKT to treat neuroses, anxiety, or other psychogenic problems remains inadequate, recent small-scale research has revealed that it is effective for depression, anxiety, and fatigue in cancer patients (Tamada et al., 2018), and for menopausal symptoms including insomnia in patients after gynecological tumor treatment (Yoshimura et al., 2018). Similar psychotropic-like effects have been demonstrated in animal experiments; KKT suppressed depression-like behavior in lipopolysaccharide-treated mice (Araki et al., 2016), and reversed anxiety-like behaviors in mice and rats induced by an inverse agonist of GABA_A_ receptors (Nishizawa and Yamashita, 1997). Anxiolytic effects of repeated KKT treatment were observed in non-stressed wild-type mice (Kuribara and Maruyama, 1996). KKT improved cognitive impairment in senescence-accelerated mice (Nishizawa et al., 1990), scopolamine-treated rats (Egashira et al., 2007), and a genetic mouse model of Alzheimer’s disease (AD) (Tohda et al., 2011). At the molecular level, chronic KKT administration to rats has been shown to modulate the binding patterns of radiolabeled ligands for neurotransmitter receptors in the brain including acetylcholine (ACh), glutamate, dopamine (DA), serotonin (5-HT), and gamma-aminobutyric acid (GABA) receptors, which are all implicated in cognitive function and/or emotional circuits (Hayashi et al., 1994; Ishihara et al., 1994; Yamada et al., 1994). Nevertheless, these reports lacked behavioral analyses. Based on the Kampo concept, KKT is categorized within the “Ginseng Root and Astragalus Root drug group” as it contains two tonic components, *Ginseng Radix* and *Astragali Radix* in its formula, both of which have various anti-aging-like effects and enhance the adaptability to stress in animal models (Choi, 2008; Liu et al., 2017; Yang et al., 2017). These effects highlight the therapeutic potential of KKT for age-related decline in physiological functions. Based on these clinical and animal experiments, KKT is a promising candidate to treat cognitive and mental frailty. Nevertheless, the pharmacological effects of KKT on behavioral characteristics in naturally aged animals has yet to be reported.

Therefore, we aimed to investigate the effects of KKT on various behavioral properties in aged wild-type C57BL/6J mice, including affective behaviors and cognitive functions. We used 18-month-old mice as our aged model, since changes in many aging-associated biomarkers (e.g. percentage of packed erythrocytes, concentration of CD4+ and CD8+ T cells, amount of collagen cross-linking in the tendons, tissue repair speed, blood glucose and insulin levels, etc.) are reportedly detected at this age (Flurkey et al., 2007). Moreover, mice have been shown to exhibit some behavioral deficits including decline in grip strength, rotarod performance, working memory, hedonic responses, and motivational behaviors by this age (Malatynska et al., 2012; Shoji and Miyakawa, 2019). To objectively evaluate diverse outcome measures, we introduced a fully automated behavioral assay system, IntelliCage, in which resident mice tagged with radio frequency identification devices (RFIDs) were group-housed and automatically imposed with a sequence of behavioral tasks (Galsworthy et al., 2005). Notably, the pharmacological validity of this high throughput assay system has been confirmed for drugs used to treat central nervous system (CNS) impairments, such as cognitive dysfunction and depression-like behaviors in mice (Branchi et al., 2010; Sekiguchi et al., 2011). To address the effects of KKT on age-related behavioral properties, we assembled and imposed a battery of tests on young and aged mice that measured their diurnal rhythms, hedonic responses, perseveration, and place learning ability in the IntelliCage.

## Materials and Methods

### Drugs

#### Diazepam

Diazepam was purchased from Wako Pure Chemical Industries (Tokyo, Japan). It was suspended in 10 mL of saline, and 5% Tween 80 was added just prior to use. The solution was intraperitoneally administered to mice.

#### Kamikihito (KKT)

The formula to produce 5 g of dried extract of KKT comprised the following 14 components: *Astragali Radix* (3 g, root of *Astragalus propinquus* Schischkin or *Astragalus mongholicus* Bunge), *Bupleuri Radix* (3 g, root of *Bupleurum falcatum* L.), *Ziziphi Semen* (3 *g*, seed of *Ziziphus jujuba* var. *spinosa* [Bunge] Hu ex H.F.Chow), *Atractylodis Lanceae Rhizoma* (3 *g*, rhizome of *Atractylodes lancea* [Thunb.] DC. or *Atractylodes chinensis* [Bunge] Koidz.), *Ginseng Radix* (3 g, root of *Panax ginseng* C.A.Mey.), *Poria* (3 g, sclerotium of *Wolfiporia cocos* Ryvarden et Gilbertson), *Longan Arillus* (3 g, arillus of *Dimocarpus longan* Lour.), *Polygalae Radix* (2 *g*, root of *Polygala tenuifolia* Willd.), *Gardeniae Fructus* (2 g, fruit of *Gardenia jasminoides* J.Ellis), *Ziziphi Fructus* (2 g, fruit of *Ziziphus jujuba* var. *inermis* [Bunge] Rehder), *Angelicae Radix* (2 g, root of *Angelica acutiloba* [Siebold & Zucc.] Kitag. or *Angelica acutiloba* var. *sugiyamae* Hikino), *Glycyrrhizae Radix* (1 g, root of *Glycyrrhiza uralensis* Fisch. ex DC. or *Glycyrrhiza glabra* L.), *Zingiberis Rhizoma* (1 g, rhizome of *Zingiber officinale* Roscoe), and *Saussureae Radix* (1 g, root of *Aucklandia costus* Falc.). All raw materials were supplied by Tsumura & Co. (Tokyo, Japan). The dry-powdered extract of KKT (lot number: 351152900 and 361095300) was produced by Tsumura & Co. Briefly, the mixture of the 14 raw materials was extracted in boiling water for 1 h, and the extract was then separated from insoluble waste. The separated extract was concentrated under reduced pressure and then spray-dried to produce the extract powder of KKT (hereafter termed “KKT”). The quality of KKT was confirmed to meet the Japanese Pharmacopoeia and our company’s standards; specifically, the following ingredients were included (with their corresponding ranges): saikosaponin b2 (0.8–3.2 mg); geniposide (27–81 mg); glycyrrhizinic acid (6–18 mg), in 5 g of KKT. All voucher specimens of the raw materials used for each lot of KKT were deposited in the herbarium of Tsumura & Co., with batch numbers (**Table S1**). For use in the elevated plus maze (EPM) test, KKT was suspended in 10 mL of distilled water immediately prior to use and was orally administered. To administer KKT without manually handling the mice in the IntelliCage experiment, KKT was suspended in tap water in the water bottles at a concentration of 1% (w/v) and placed in the cage. The bottles of KKT suspension were refreshed every 2 to 3 days.

### Animals

All mice used in this study were male and purchased from Charles River Laboratories Japan (Yokohama, Japan). Aged mice were grown to 18 months old by the vendor, and those without any apparent injuries or loss of voluntary movements were used for experimentation. All mice were maintained at a temperature of 23 ± 3°C, relative humidity of 50 ± 20%, and 12-h light-dark cycle, with *ad libitum* access to food and water unless otherwise stated. All animal experiments were conducted in accordance with the guidelines for the care and use of experimental animals of Tsumura & Co. All the experimental procedures were approved by the experimental animal ethics committees of Tsumura & Co.

### Elevated Plus Maze (EPM) Test

The maze comprised four equally sized arms (5 cm wide × 30 cm deep) that were 40 cm above the experimental table, two of which were enclosed by 20-cm-high walls and two of which were open. The apparatus was dimly illuminated (40–50 lux). During each test, a mouse was gently placed in the center area of the maze, and its exploratory behavior was monitored for 10 min by a CCD camera (Panasonic, Osaka, Japan). During this time, the time spent in the open arms (OAs), number of entries to each arm, and distance traveled in the EPM were recorded using LimeLight 2 (Actimetrics, IL) as indicators of anxiety-like behavior (more time in OAs indicates less anxiety). The percentage of OA entries was calculated as: (number of entries to OAs)/(number of entries to all four arms) × 100 (higher percentage indicates less anxiety). All mice were habituated to the rearing condition for at least 1 week before grouping, and were 8–10 weeks old on the day of the EPM test. To evaluate the effects of diazepam, mice (n = 23) were randomly divided into the following three groups based on the dose of diazepam injected: 0 mg/kg (i.e., vehicle; n = 8), 0.5 mg/kg (n = 8), and 1.0 mg/kg (n = 7). The EPM test was conducted 30 min after a single administration of either vehicle or diazepam. To examine the effects of single administration of KKT, the EPM test was performed at three different intervals (i.e., 30 min, 2 h, and 4 h) after administration. Mice (n = 95) were randomly divided based on the dose of KKT for each interval as follows: 30 min interval: 0 g/kg (n = 16), 1 g/kg (n = 16), and 2 g/kg (n = 15); 2 h interval: 0 g/kg (n = 8), 1 g/kg (n = 8), and 2 g/kg (n = 8); 4 h interval: 0 g/kg (n = 8), 1 g/kg (n = 8), and 2 g/kg (n = 8). For repeated KKT treatment, mice (n = 53) were randomly divided into the following groups of different doses: 0 g/kg (n =18), 1 g/kg (n = 17), and 2 g/kg (n = 18) groups. Mice were treated with either vehicle or KKT once daily for 7 consecutive days and were tested 24 h after the last administration, as described previously (Kuribara and Maruyama, 1996). For all experiments, the EPM test was performed between 10:00 and 16:00.

### IntelliCage experiment

#### Test Apparatus

IntelliCage (TSE Systems GmbH, Germany) is a large plastic cage (61.0 × 43.5 × 21.5 cm^3^) equipped with four triangular operant chambers (hereafter referred to as corners) (15 × 15 × 21 cm^3^). This fully automated testing apparatus enables behavioral monitoring of group-housed RFID-tagged mice in a home cage setting. The corner entrance and inner space are large enough for a single mouse to enter at a time. Each mouse is recognized by the RFID antennas at its entrance. Each corner holds two water bottles, and their nozzles are accessed through nose-poke (NP) holes with motorized access doors. The infrared beam-break response detector on each of the NP holes responds to the mouse NPs, which trigger door opening. Experimenters can flexibly program the rules for opening/closing of the doors.

#### General

Young (arrived at the facility at 7–8 weeks old) and aged (18 months old) C57BL/6J mice were assigned to either control or treatment groups (n = 8 per group, unless otherwise stated); namely, young control, young KKT-administered, aged control, or aged KKT-administered groups. Immediately after arrival from the vendor, the four groups were maintained in separate standard cages for a week before being introduced to the IntelliCage. The treatment groups received KKT in drinking water (1% w/v) throughout the experiment, including the first week in the standard cages; the control groups received regular drinking water. During the IntelliCage experiment, except for the saccharin preference and effort-based choice behavior tests, the left bottle at each corner contained regular water for the control groups; the right bottle contained 1% KKT suspension for the KKT groups. Two IntelliCages were used in the experiment: one contained the young mice; the other contained the aged mice. The experimental day (day 1: the day of transfer to the IntelliCage) was defined as the period starting from the beginning of the light phase to the end of the following dark phase. Each mouse’s behavioral responses were recorded through the various sensor devices of the IntelliCage, associated with the animal’s ID, and used to analyze each spontaneous/cognitive behavioral index. Cages were cleaned once a week.

#### Basal Activity (Days 2–15)

Door opening was triggered by a NP; this condition remained as such for the rest of the experiment (unless otherwise stated). A single NP opened the door for 4,000 ms to allow the control group to drink regular water and treatment group to drink KKT suspension. Corner visits and NPs for 2 weeks were recorded and analyzed as indices of basal activity. Two aged mice (one in the control and one in the KKT group) died during this period; the data from these individuals were removed from analyses. One week before starting the subsequent place learning and behavioral flexibility test, substitute individuals that had been maintained in separate standard cages with either control water or KKT suspension were recruited into the aged control and aged KKT groups, respectively, in the IntelliCage.

#### Place Learning and Behavioral Flexibility Test (Days 17–29)

In this test, each mouse was assigned one of two pairs of diagonally positioned corners as “correct corners”. The mice had to shuttle between the two correct corners to obtain water or KKT solution as a reward. Drinking was not permitted at the remaining corners until the rule was switched. When a mouse performed a NP at one of the correct corners, the door opened for 2,000 ms to permit liquid intake, with the following restrictions: the door opened only once per visit, and a mouse was not permitted to obtain multiple rewards in a row at the same corner. Success (receiving a reward by a direct move from the previously rewarded corner to the other correct corner) and failure (visiting one of the two non-reward corners) responses were integrated into Wald’s sequential probability ratio test statistics to calculate each mouse’s probability of success. Visits without NPs were omitted from the calculation. Whenever a mouse’s performance reached the set upper threshold, the assignment of the reward and non-reward corners was reversed (reversal learning). The number of trials (the sum of the above mentioned success and failure responses) required to reach the upper threshold for rule reversal was used to assess the efficiency of learning and behavioral flexibility; the upper threshold was set at 30% for the first session and 60% for the second session. The chance level of this task was 20%. The significance level for the acceptance of each threshold was 0.05. No set task period was imposed, such that each mouse performed the task self-paced. Therefore, the timing of the rule reversal varied for each mouse. For both sessions, the task started at 20:00 and lasted for 24 h × 6 days, with a 24-hour interval between each session during which no set task was imposed.

#### Saccharin Preference and Effort-Based Choice Behavior Test (Days 31–43)

This test was designed to evaluate the effects of KKT on hedonic capacity and motivation in the IntelliCage. The interior space of the IntelliCage was divided into two compartments by an acrylic divider. The control and KKT groups were separately maintained and tested in one of the compartments, and allowed to access two corners each. When a mouse entered a corner, both right and left doors of the NP hole opened simultaneously, giving the mice access to two bottles for the tasting trial. Control groups were able to drink from one bottle containing regular water or one containing 0.1% saccharin in tap water; KKT groups were able to drink from either one bottle containing KKT solution or one containing 0.1% saccharin in KKT solution. After 1,000 ms of drinking, both doors closed. After the tasting trial, the chosen door re-opened for 2,000 ms per NP. The number of re-openings per visit at either water/KKT solutions or 0.1% saccharin was recorded as an index of saccharin preference. The positions of the 0.1% saccharin bottles were switched daily. The first 2 days (session 1) were conducted as described above as a simple saccharin preference test. For the following 2 days (session 2), the KKT group could choose between regular water vs. 0.1% saccharin in regular water, instead of KKT solution. Both sessions started at 20:00. Motivation to drink sweet solutions was assessed using a similar experimental set up as that for sessions 1 and 2, except that it required two NPs to re-open the chosen door after tasting trials to obtain a reward. After 50 re-openings (the regular water/KKT solution side and 0.1% saccharin side combined), the number of NPs required to re-open the 0.1% saccharin increased incrementally by one up to nine NPs, while the number of NPs required to re-open the regular water/KKT solution side remained as two; i.e., mice were subjected to an increasing workload to drink 0.1% saccharin compared to that for regular water/KKT solution. The effort-based task started at 20:00 and continued for 24 h × 4 days.

### Statistical Analyses

Statistical significance was defined as P < 0.05. All two-group comparisons were performed using unpaired t-tests. Data of two variables were analyzed with two-way analysis of variance (two-way ANOVA), and chronological data were analyzed using two-way repeated measures ANOVA. Multiple comparisons were performed using Dunnett’s test or Bonferroni’s test. All statistical methods used are specified in the figure legends. All data were analyzed with GraphPad Prism 7 (GraphPad Software, San Diego, CA).

## Results

### Anxiolytic Effects of KKT in the Elevated Plus Maze (EPM) Test

**Figure 1:**
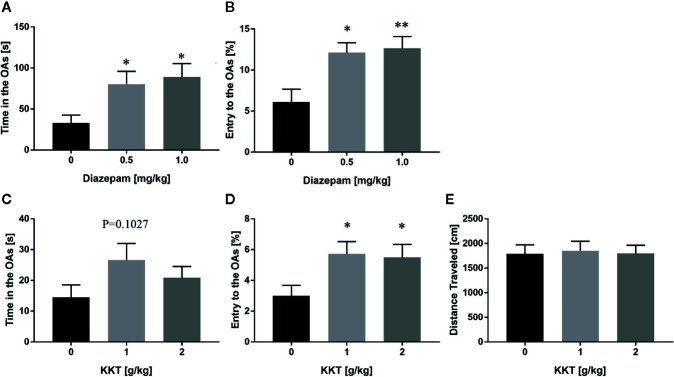
Anxiolytic effects of KKT on C57BL/6J mice in the conventional EPM. **(A, B)** Effect of single administration of diazepam on time spent **(A)** and percentage of entries **(B)** in the OAs (n = 7–8). **(C–E)** Effects of repeated KKT treatment on time spent in the OAs **(C),** the percentage of OA entries **(D),** and total distance traveled in all arms **(E)** (n = 17–18). All data are presented as mean ± SEM. *: P < 0.05; **: P < 0.01 (versus the vehicle-administered group), Dunnett’s test.

In a previous study, repeated KKT treatment was shown to decrease anxiety-like behaviors in mice in the EPM test (Kuribara and Maruyama, 1996). To recapitulate the anxiolytic effects and determine the effective dosage of KKT, we established an in-house EPM setting, and found that a single administration of diazepam, a known anxiolytic, caused an increase in the time spent in the OAs (0.5 mg/kg: q = 2.447, df = 20, P = 0.0437; 1.0 mg/kg: q = 2.805, df = 20, P = 0.0204) and percentage of OA entries (0.5 mg/kg: q = 3.097, df = 20, P = 0.0107; 1.0 mg/kg: q = 3.257, df = 20, P = 0.0075) compared to that of the vehicle-administered group (**Figures 1A, B**). In this setting, repeated KKT treatment resulted in a trend for extending the time spent in the OAs at a dose of 1 g/kg, but not 2 g/kg (1 g/kg: q = 1.947, df = 50, P = 0.1027; 2 g/kg: q = 1.037, df = 50, P = 0.4839), and a significant increase in the percentage of OA entries at both 1 and 2 g/kg doses (1 g/kg: q = 2.472, df = 50, P = 0.0315; 2 g/kg: q = 2.29, df = 50, P = 0.0485) (**Figures 1C, D**). The distance traveled in the EPM was not changed by either dose of KKT (1 g/kg: q = 0.2488, df = 50, P = 0.9560; 2 g/kg: q = 0.03185, df = 50, P = 0.9993) (**Figure 1E**). Conversely, a single administration of KKT had no effect on exploratory behaviors in the EPM at any time point after administration at either dose (**Supplementary Figure 1**). These data demonstrated that KKT exerted anxiolytic effects as reported previously and that repeated, but not single, treatment of C57BL/6J mice was effective at doses ranging from 1–2 g/kg.

To achieve an effective dose without manually handling mice in the IntelliCage, water bottles filled with KKT suspension were presented to both age groups. Water consumption per body weight of C57BL/6J mice was approximately 0.13 mL/g/day and was comparable between young and aged individuals in our previous study (Oizumi et al., 2019). Therefore, KKT concentration was set to 1% (w/v), whereby the daily intake of KKT was estimated to be approximately 1.3 g/kg.

### Basal Activities in the IntelliCage

**Figure 2:**
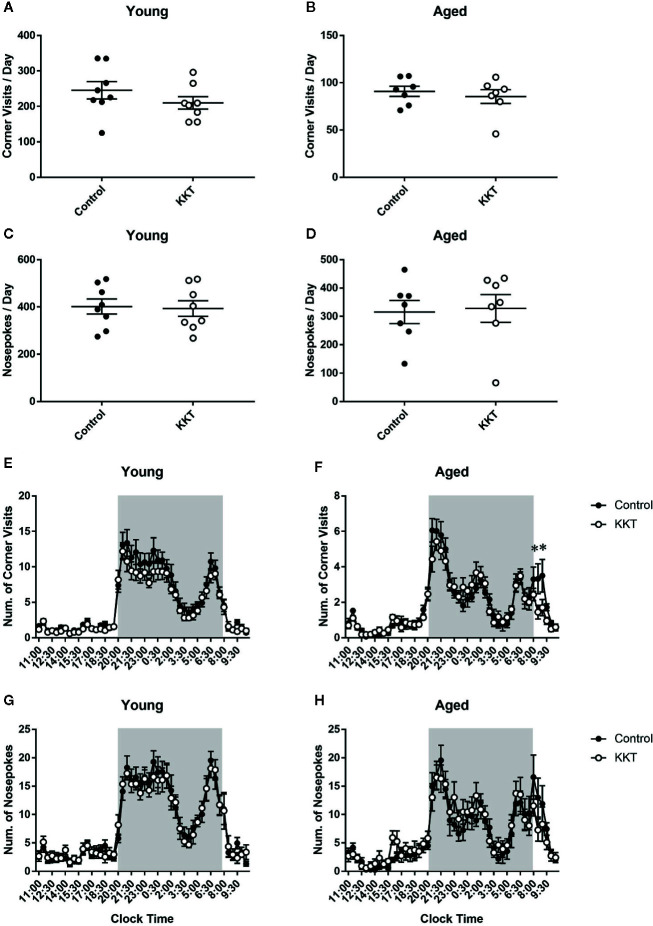
Effects of KKT on basal activities. **(A–D)** Scatterplot depicting the number of corner visits (young: **(A)**; aged **(B)** and NPs (young: **(C)**; aged **(D)** per day are presented as the mean ± SEM. Statistical significance was analyzed using unpaired t-tests. **(E–H)** The diurnal variation of corner visits (young: **(E)**; aged: **(F)** and NPs (young: **(G)**; aged **(H)** logged every 30 mins. Shaded area indicates the dark period (20:00–8:00). Data are expressed as mean ± SEM. n = 8 (young) and 7 (aged), *: P < 0.05, Bonferroni’s multiple comparison.

During days 2–15 of the IntelliCage experiment, in which no intentional tasks were imposed on resident mice, KKT did not alter the number of corner visits per day (young: t = 1.183, df = 14, P = 0.2563; aged: t = 0.6173, df = 12, P = 0.5486) or NPs per day (young: t = 0.1921, df = 14, P = 0.8504; aged: t = 0.2027, df = 12, P = 0.8427) in age-matched comparisons (**Figures 2A–D**). In young mice, diurnal variations in the number of corner visits and NPs for every 30 minutes averaged over 2 weeks was significantly affected by time (corner visits: F [47, 658] = 60.46, P < 0.0001; NPs: F [47, 658] = 46.95, P < 0.0001), but not treatment (corner visits: F [1, 14] = 1.401, P = 0.2563; NPs: F [1, 14] = 0.03692, P = 0.8504). No significant time × treatment interaction was detected (corner visits: F [47, 658] = 0.8094, P = 0.8152; NPs: F [47, 658] = 0.4267, P = 0.9997) (**Figures 2E, G**). In contrast, two way repeated measures ANOVA of the number of corner visits of aged mice indicated a significant time × treatment interaction (F [47, 564] = 1.611, P = 0.0075) with a significant main effect of time (F [47, 564] = 36.89, P < 0.0001) but not treatment (F [1, 12] = 0.381, P = 0.5486) (**Figure 2F**). Post-hoc multiple comparison revealed that the number of corner visits in the aged KKT group was lower than that in the controls at two intervals from 8:00 to 9:00 a.m. (8:00–8:30: t = 3.714, df = 576, P = 0.0108; 8:30–9:00: t = 3.552, df = 576, P = 0.0198) (**Figure 2F**). The number of NPs of aged mice was affected only by time (time: F [47, 564] = 22.23, P < 0.0001; treatment: F [1, 12] = 0.0411, P = 0.8427), with no significant time × treatment interaction (F [47, 564] = 1.161, P = 0.2207) (**Figure 2H**). These results suggested that KKT accelerated decreased activity just after the beginning of the light phase in aged but not young mice, without affecting total activity levels or liquid consumption. It is known that residents in the IntelliCage do not access the four corners evenly but have a slight preference for certain corners (Endo et al., 2011). KKT had no effect on either the percentage of corner visits or NPs in rank-matched comparisons of the respective corners in both age groups (**Supplementary Figures 2A–D**). In addition, successive movement patterns towards the four corners were classified into four modes (i.e., diagonal, short side, long side, and re-entry moves), none of which were affected by KKT treatment in both age groups (**Supplementary Figures 2E, F**). These data indicated that KKT did not alter basal movement patterns in either age group.

### Effects of KKT on Place Learning and Behavioral Flexibility

**Figure 3:**
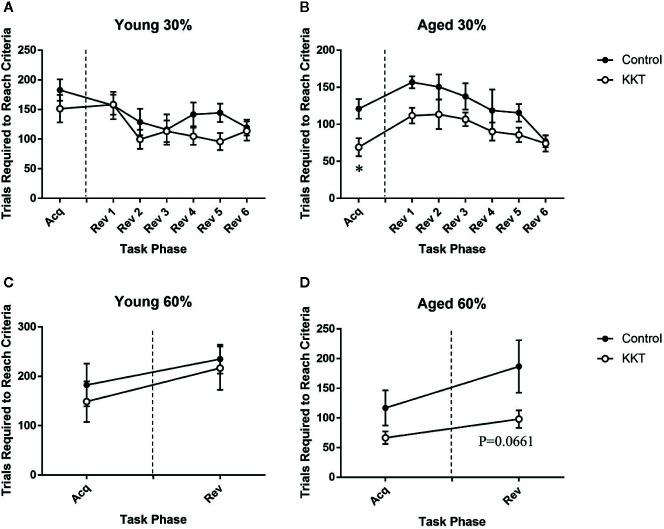
Effects of KKT on cognitive functions. **(A, B)** Trials required to reach the threshold of 30% success probability in young **(A)** and aged **(B)** mice. Unpaired t-tests were used for statistical analysis of the acquisition phase (*: P < 0.05). Data of serial reversal sessions were analyzed by two-way repeated measures ANOVA followed by Bonferroni’s multiple comparison (n = 8). **(C, D)** Trials required to reach the threshold of 60% success probability in young **(C)** and aged **(D)** mice. Data of acquisition and reversal sessions were analyzed with unpaired t-tests. n = 8 (young control), 8 (young KKT), 7 (aged control), and 8 (aged KKT). Note that one mouse of the aged control group failed to reach threshold during the acquisition phase. All data are expressed as mean ± SEM.

Following the assessment of basal activity, mice were subjected to a place learning and behavioral flexibility task in which two diagonally positioned corners were set as “correct corners” at which they could drink from water bottles. The other corners were set as “incorrect corners” at which mice were unable to drink. If a mouse succeeded in drinking at one correct corner, it was able to subsequently drink only at the other correct corner. Each mouse had to learn to shuttle diagonally between the correct corners to continuously obtain liquid. The number of trials required to reach the learning threshold of the calculated success probability (30%) was not altered by KKT treatment in the young group (t = 1.071, df = 14, P = 0.3025) (**Figure 3A**), whereas that in the aged mice was significantly reduced by KKT treatment (t = 2.873, df = 14, P = 0.0123) (**Figure 3B**). The positions of correct and incorrect corners were interchanged immediately after the learning acquisition scores for each individual reached the threshold. This reversal task was repeated each time a mouse reached the threshold. During the reversal session, the scores of the young animals were not influenced by KKT treatment (F [1, 14] = 2.348, P = 0.1477) or task phase (F [5, 70] = 1.833, P = 0.1176), and there was no significant treatment × phase interaction (F [5, 70] = 0.7186, P = 0.6116) (**Figure 3A**). In contrast, the learning scores of the aged groups were significantly affected by both treatment (F [1, 14] = 9.493, P = 0.0081) and task phase (F [5, 70] = 4.814, P = 0.0008), with no significant interaction between treatment and task phase factors (F [5, 70] = 0.4857, P = 0.7858), although *post hoc* multiple comparisons failed to detect significant changes at any phase points of the reversal sessions (**Figure 3B**). Subsequently, the learning threshold was raised from 30% to 60% success probability to assess the effects of KKT on more difficult learning tasks. Similar to previous sessions, KKT failed to elevate the learning performance of young mice in either the acquisition (t = 0.5636, df = 14, P = 0.5820) or reversal sessions (t = 0.3464, df = 14, P = 0.7342) (**Figure 3C**). The acquisition efficiency of aged mice was also unchanged by KKT (t = 1.683, df = 13, P = 0.1162), but there was a trend for improvement in performance in the reversal session (t = 2.007, df = 13, P = 0.0661), although this did not reach statistical significance (**Figure 3D**). These data suggested that KKT moderately enhanced cognitive functions in the initial acquisition of shuttle moving rules and in subsequent serial reversal sessions specifically in aged mice.

### Decrease in Perseverative NPs at Correct Corners During Place Learning

**Figure 4:**
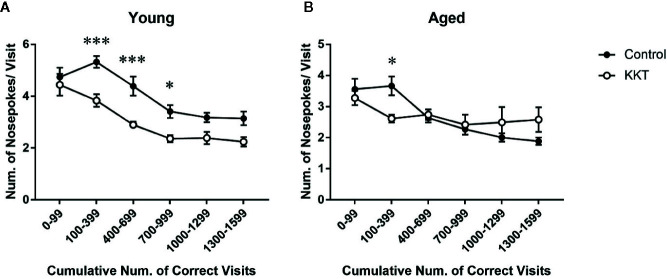
Effects of KKT on perseverative NPs of young **(A)** and aged **(B)** mice during the learning sessions. Data were collected every 300 corner visits except for the initial part of learning during which data were measured in 100 visits. All data are presented as mean ± SEM. n = 8, two-way repeated measures ANOVA followed by Bonferroni’s multiple comparison (*: P < 0.05; ***: P < 0.001).

Although only one NP per corner visit was required to obtain liquid, mice are prone to perform multiple NPs in a visit during place learning and behavioral flexibility tests (Endo et al., 2012). Such superfluous NPs conducted before obtaining rewards, which no longer yield additional rewards, are regarded as perseverative behavior and considered to reflect compulsivity and inflexibility of behavioral responses (Endo et al., 2012; Carli and Invernizzi, 2014). In the young mice, the number of NPs per visit to the correct corners was significantly influenced by both treatment (F [1, 14] = 12.25, P = 0.0035) and cumulative number of visits (F [5, 70] = 45.12, P < 0.0001), with a significant interaction between the main factors (F [5, 70] = 2.996, P = 0.0165) (**Figure 4A**). Post-hoc analyses indicated that the number of NPs per visit in the KKT group was significantly lower than that in the control group during the phases of 100–999 visits to the correct corners (100–399: t = 3.988, df = 84, P = 0.0009; 400–699: t = 3.987, df = 84, P = 0.0009; 700–999: t = 2.805, df = 84, P = 0.0375) (**Figure 4A**). Similarly, the number of NPs by the aged mice was affected by the number of corner visits (F [5, 70] = 11.26, P < 0.0001) but not treatment (F [1, 14] = 0.004124, P = 0.9497), and the interaction was statistically significant (F [5, 70] = 4.331, P = 0.0017) (**Figure 4B**). Subsequent multiple comparisons revealed reduced NPs per visit in the KKT group at an interval of 100–299 visits (t = 2.736, df = 84, P = 0.0455) compared with those in the control group (**Figure 4B**). These results revealed that perseverative NPs, which were observed mainly during the early part of the learning period, were alleviated by KKT in both age groups.

### Effect of KKT on Sweet Preference

**Figure 5:**
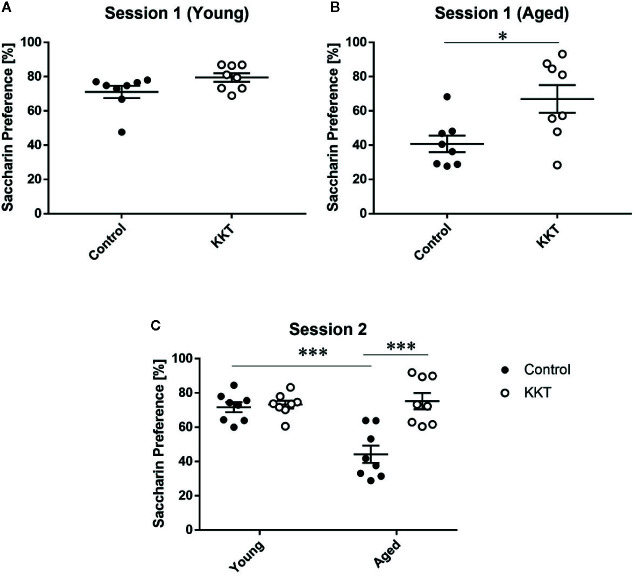
Effects of KKT on sweet preference. **(A, B)** The percentage of saccharin preference of young **(A)** and aged **(B)** mice in session 1. Note that 1% KKT suspension was used as the solvent for saccharin in the KKT groups. Statistical significance was analyzed using unpaired t-tests (*: P < 0.05). **(C)** The percentage of saccharin preference was calculated for session 2 in which tap water was used as the solvent for saccharin for both control and KKT groups. Statistical analysis was performed by two-way ANOVA followed by Bonferroni multiple comparison (***: P < 0.001). Scatterplot data are presented as mean ± SEM (n = 8).

Mice have an innate preference for sweet substances, which is reduced by various experimental stimuli, including stress exposure (Willner, 2016; Scheggi et al., 2018). Reduced preference for sweet solutions over vehicle liquid is thought to reflect a reduced ability to experience pleasure in human depressive symptoms and can be reversed by chronic treatment with antidepressants. This phenomenon is thus regarded as an indicator of anhedonia in rodents (Willner, 2016; Scheggi et al., 2018). To examine the effects of KKT on sweet preference, we employed the two-bottle choice paradigm using vehicle solution and 0.1% saccharin dissolved in vehicle, where water and 1% KKT suspension were used as the vehicle for control and KKT groups, respectively (session 1). The percentage saccharin preference of young mice did not significantly differ between control and KKT groups (t = 1.933, df = 14, P = 0.0737) (**Figure 5A**). In the aged groups, saccharin preference was significantly increased following KKT treatment (t = 2.77, df = 14, P = 0.0150) (**Figure 5B**). To exclude the possibility that the taste of KKT directly affected the attractiveness of saccharin taste in mice, we replaced 1% KKT suspension with tap water as the vehicle for saccharin in the KKT groups in the subsequent session (session 2). In session 2, we tested the effects of KKT treatment as well as age on saccharin palatability. Two way ANOVA detected significant main effects (treatment: F [1, 28] = 17.09, P = 0.0003; age: F [1, 28] = 10.48, P = 0.0031), with a significant interaction between treatment and age factors (F [1, 28] = 14.04, P = 0.0008) (**Figure 5C**). Similar to that in session 1, post-hoc multiple comparisons revealed that the saccharin preference of the aged KKT group was significantly higher (t = 5.573, df = 28, P < 0.0001) and that of the young KKT group remained unchanged (t = 0.2733, df = 28, P > 0.9999) compared with that of age-matched controls (**Figure 5C**). The saccharin preference of aged controls was significantly lower than that of young controls (t = 4.939, df = 28, P = 0.0002) (**Figure 5C**). These findings indicated that sweet preference declined with increasing age and that KKT specifically augmented consummatory hedonic responses in aged mice.

### Increase in Motivated Behaviors by KKT Treatment

**Figure 6:**
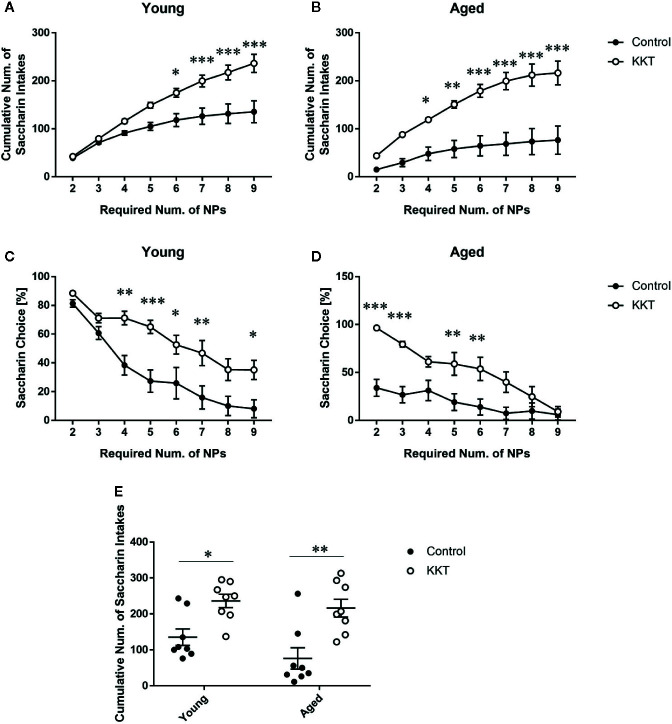
Effects of KKT on effort-based decision making. **(A–D)** Effects of KKT on cumulative saccharin intake attained by young **(A)** and aged **(B)** groups, and saccharin preference in young **(C)** and aged **(D)** mice during a progressive ratio task. Data are expressed as mean ± SEM (n = 8). *: P < 0.05; **: P < 0.01; ***: P < 0.001, Bonferroni’s multiple comparison following two-way repeated measures ANOVA. **(E)** Age-related effects of KKT on total saccharin solution volume obtained. Scatterplot data are presented as mean ± SEM (n = 8). Statistical analysis was conducted with two-way ANOVA followed by Bonferroni multiple comparison (*: P < 0.05; **: P < 0.01).

After the second session of the sweet preference test, the resident mice were subjected to an effort-related choice behavior task in which the number of NPs required to obtain the saccharin solution, but not the vehicle, was automatically increased in a stepwise manner. In both age groups, cumulative saccharin intake was significantly influenced by KKT treatment (young: F [1, 14] = 12.75, P = 0.0031; aged: F [1, 14] = 21.41, P = 0.0004) and effort required (young: F [7, 98] = 85.65, P < 0.0001; aged: F [7, 98] = 35.01, P < 0.0001), with significant interactions (young: F [7, 98] = 10.74, P < 0.0001; aged: F [7, 98] = 8.284, P < 0.0001) (**Figures 6A, B**). Post-hoc analyses indicated significantly increased attainment of saccharin solution after KKT treatment at certain phases in age-matched comparisons (**Figures 6A, B**). Similar to cumulative saccharin intake, saccharin preference was significantly affected by KKT treatment (young: F [1, 14] = 11.63, P = 0.0042; aged: F [1, 14] = 13.46, P = 0.0025) and task phase (young: F [7, 98] = 53.03, P < 0.0001; aged: F [7, 98] = 23.85, P < 0.0001) at both ages, with significant interactions between treatment and task phase factors (young: F [7, 98] = 3.122, P = 0.0051; aged: F [7, 98] = 5.939, P < 0.0001) (**Figures 6C, D**). Increased saccharin preferences in the KKT groups were detected by post-hoc multiple comparisons at certain phases in both age groups relative to those in age-matched controls (**Figures 6C, D**). Furthermore, we directly examined age-related differences in KKT effects on motivated behaviors. Two way ANOVA of cumulative saccharin intake illustrated a significant main effect of treatment (F [1, 28] = 24.61, P < 0.0001) but not age (F [1, 28] = 2.644, P = 0.1151), with no significant treatment × age interaction (F [1, 28] = 0.6526, P = 0.4260) (**Figure 6E**). Post-hoc analyses indicated that KKT-administered mice of both age groups ingested more saccharin solution than did their age-matched controls (young: t = 2.936, df = 28, P = 0.0394; aged: t = 4.079, df = 28, P = 0.0020), with no significant difference in saccharin intake in treatment-matched comparisons (water control: t = 1.721, df = 28, P = 0.5777; KKT: t = 0.5785, df = 28, P > 0.9999) (**Figure 6E**). These results demonstrated that KKT enhanced decisions for taking high-effort choices independent of mouse age.

## Discussion

In the present study, we demonstrated the following effects of KKT on C57BL/6J mouse behavior: (1) repeated, but not single, administration of KKT produced anxiolytic effects in young mice; (2) KKT accelerated activity decline in aged, but not young, mice at the beginning of the light phase; (3) KKT enhanced place learning and behavioral flexibility performance in aged mice; (4) perseverative behaviors were suppressed by KKT in both young and aged mice; (5) KKT increased sweet preference only in aged mice; and (6) KKT enhanced motivated behaviors in both age groups.

In agreement with previous findings (Kuribara and Maruyama, 1996), we demonstrated that repeated KKT treatment increased the percentage of OA entries (**Figure 1D**) but not the time spent in the OAs of the EPM, another proxy of anxiety in mice (**Figure 1C**), suggesting that the anxiolytic effects of KKT may be mild. To confirm the generalizability of the anxiolytic effects of KKT, further pharmacological studies are required using other behavioral tests based on various fear/reward conflicts, especially, the intensity, type, context, and duration of fear exposure. Given that single administration of KKT failed to suppress anxiety behaviors in mice, alteration of protein expression patterns in the brain by repeated KKT treatment may be required for its anxiolytic effects. Indeed, repeated administration of *Bupleuri Radix* (BR), a component of the KKT formula, significantly suppressed stress-induced upregulation of tyrosine hydroxylase in the locus coeruleus, which is the main noradrenergic center in the brain, thereby preventing anxiety behavior in the EPM (Lee et al., 2012) and suggesting possible long-term alterations in neurotransmitter systems by chronic KKT treatment. In contrast, acute anxiolytic effects have recently been reported following single administration of rutin, a major flavonoid contained in *Ziziphi Fructus*, which is mediated partly through GABAergic pathways (Hernandez-Leon et al., 2017). In addition, *Zingiberis Rhizoma* (ZR) extract, another component of KKT, has been reported to exert acute anxiolytic effects (Vishwakarma et al., 2002), although the underlying mechanisms are uncertain. Collectively, the anxiolytic effects of KKT may be coordinately induced by long-lasting molecular changes in the brain, and mild/acute effects on certain neurotransmitter systems.

In the IntelliCage experiments, many of the behavioral tasks were designed based on thirst in resident mice. During the first 2 weeks in the IntelliCage, the total number of corner visits and NPs per day was similar in age-matched comparisons (**Figures 2A–D**), confirming that KKT had no effect on thirst or drinking behaviors per se. However, the diurnal variation in aged, but not young, mice was altered by KKT treatment; specifically, the aged KKT group exhibited fewer corner visits than did the control group during the early part of the light phase (**Figure 2F**), suggesting that KKT may accelerate sleep onset in aged mice. Of numerous neural networks relevant to the balance of arousal and sleep (Sakurai, 2007; Scammell et al., 2017), the wake-promoting orexinergic neurons are noteworthy as potential targets of KKT, as spinosin, a major ingredient of *Ziziphi Semen* (ZS), reduces the activation of orexinergic neurons in the lateral hypothalamic area (Zhang et al., 2019). Regarding the influence of aging, older mice exhibited a decrease in rapid eye movement (REM) sleep during the early part of the light phase compared to that in younger mice (Wimmer et al., 2013; Soltani et al., 2019). In this regard, reduced quality of REM sleep in a rat AD model, reflected by a decrease in theta power density during REM sleep, was ameliorated by the Kampo medicine sansoninto, which includes ZS as a major component (Moriyama et al., 2017). Collectively, these results suggest that KKT may accelerate the sleep onset or potentiate the quality of sleep of aged mice *via* ingredients of ZS. To confirm this, electroencephalogram recordings should be performed to directly assess the effect of KKT on their sleep.

Place discrimination and reversal learning assessed in the IntelliCage system are affected by pharmacological or genetic manipulation of various neural circuits such as dopaminergic, cholinergic, and serotonergic pathways (Sekiguchi et al., 2011; Macpherson et al., 2016; Pelsőczi and Lévay, 2017; Marwari and Dawe, 2018; Marwari and Dawe, 2019). Among these neurotransmitters, DA signaling is of particular interest, as tenuifolin, an ingredient of *Polygalae Radix* (PR), was shown to elevate DA levels in the hippocampus of aged and dysmnesia mice alongside improving impaired learning performance (Zhang et al., 2008). In addition, several ingredients of PR have inhibitory activities against DA transporter and DA metabolism-related enzymes, including monoamine oxidase B and catechol-*O*-methyltransferase (Yamada et al., 2018). The Kampo medicine, ninjin’yoeito, which shares six formula components with KKT, including PR, reversed the reduction in goal-directed behavior in a mouse model of apathy *via* the activation of D2 receptors (Yamada et al., 2018). With regard to aging effects on the relationship between DA and cognition, aged rodents have attenuated dopaminergic transmission in the prefrontal cortex (PFC) accompanied with impaired reversal learning performance, which is effectively improved by the infusion of a D1 receptor agonist into the orbitofrontal cortex (Mizoguchi et al., 2009). As KKT enhanced cognitive function exclusively in aged mice in the current study (**Figure 3**), its effects may be mediated by the normalization of altered dopaminergic pathways in aged individuals. In contrast, perseverative nose-poking behavior, which was reduced by KKT in both young and aged groups during the learning period (**Figure 4**), was not alleviated by increased DA signaling in a 5-choice reaction time task (Carli and Invernizzi, 2014). Conversely, increased DA transmission by systemic administration of d-amphetamine augmented perseverative behavior (Baunez and Robbins, 1999), and systemic antagonism of D2 receptors by haloperidol suppressed perseverative NPs induced by the inhibition of NMDA receptors in the PFC (Baviera et al., 2008). Perseverative over-responding was alleviated by infusion of a 5-HT_1A_ receptor agonist into the PFC (Carli et al., 2006). In this respect, although it is unknown whether KKT exerts effects on 5-HT_1A_ receptors, partial agonists for 5-HT_1A_ receptors exist in the ZR extract (Nievergelt et al., 2010), raising the possibility of KKT-induced alteration of serotonergic transmission in the current study. Collectively, the effects of KKT on cognition and flexibility in mice are likely to be mediated by the coordinated orchestration of multiple neural pathways rather than a single neurotransmitter system. In this regard, its effect on serotonergic and dopaminergic transmission should be examined in future studies.

In young mice, KKT enhanced effortful NPs for saccharin without influencing basal appetite for it (**Figures 5A, C**), indicating that KKT increased motivation in young mice. In the aged group, KKT elevated motivated behaviors and improved attenuated sweet preference (consummatory anhedonia) (**Figures 5B, C**). Decreased sweet preference often develops after chronic stress exposure in rodents and can be ameliorated by repeated treatment with typical antidepressants, i.e., serotonin selective reuptake inhibitors (SSRIs), serotonin and noradrenaline reuptake inhibitors (SNRIs), and tricyclic antidepressants (Millan et al., 2001; Montgomery et al., 2008). Similar to stress-induced anhedonia, age-related decline in sweet preference was reportedly abated by chronic treatment with a tricyclic antidepressant, imipramine, whose mechanism of action predominantly involves the inhibition of serotonin and noradrenaline transporters, implying that decreased sweet preference in aged mice is psychogenic and potentially models elderly depression (Malatynska et al., 2012). ?>Thus, the effects of KKT on consummatory anhedonia in aged mice may have commonalities with the aforementioned typical antidepressants. It is noteworthy that chronic treatment with BR extract results in antidepressant-like effects in immobility time in the tail suspension test *via* the activation of serotonergic and noradrenergic systems, implying pharmacological similarity between KKT and typical antidepressants (Kwon et al., 2010).

SSRIs generally provoke a decrease in motivated behaviors for reward. This is mediated by the inhibitory effect of increased 5-HT on dopaminergic neurons in the ventral tegmental area (Sanders et al., 2007; Browne et al., 2019). Similar reductions in motivation are observed with SNRIs (Stromberg et al., 2002; Simon O’Brien et al., 2011). Based on these findings, the pharmacological mechanisms underlying the pro-motivational effects of KKT, at least in young mice, are not attributable to the global elevation in 5-HT and noradrenaline caused by typical antidepressants. Instead, the mesolimbic dopaminergic system may be the target of the hedonic-related effects of KKT, given that the effort-related choice behavior task paradigm used in this study is very sensitive to dopaminergic alterations (Scheggi et al., 2018). Notably, low doses of DA receptor antagonists have been shown to cause a reduction in lever-pressing for high reward, which is in turn recovered by an atypical antidepressant, bupropion, which increases DA levels by inhibiting the DA transporter (Nunes et al., 2013; Pardo et al., 2015). As mentioned, the effect of PR, which is a component of KKT, on DA metabolism has been indicated, supporting the possibility that KKT may enhance motivated behaviors (at least in young mice) by activating the mesolimbic DA system. Further pharmacological and histological studies are required to investigate whether KKT directly potentiates dopaminergic pathways. Moreover, it remains to be determined whether the increase in effort-related NPs in KKT-treated aged mice is attributable to improved hedonic capacity or elevated motivation.

In light of the concept of Kampo formulation, KKT is empirically categorized into the “Ginseng Root and Astragalus Root drug group”, which is characterized by two tonic components, *Ginseng Radix* (GR) and *Astragali Radix* (AR). These components not only reinforce and recover weakened body conditions, but also exhibit anti-aging properties such as beneficial effects on anti-oxidation, the immune system, cardiovascular function, cognitive function, and adaptability to stress (Choi, 2008; Liu et al., 2017; Yang et al., 2017). Among these diverse functions, anti-oxidant effects are a major anti-aging related property of both GR and AR. In particular, GR treatment in aged rats has been shown to restore the age-related decline in anti-oxidant enzymes including superoxide dismutase (SOD), catalase, glutathione peroxidase (GSH-Px), and glutathione reductase, thereby decreasing oxidative damage in organs (Ramesh et al., 2012a; Ramesh et al., 2012b). Upregulation of anti-oxidant capacity by GR has been confirmed in a randomized placebo controlled clinical trial (Kim et al., 2011). Similarly, administration of AR in rats elevates the activity of SOD, catalase, and GSH-Px, leading to a decrease in reactive oxygen species (Cao et al., 2014). GR has also been reported to have anti-inflammatory effects (Yang et al., 2017). Notably, a recent study revealed that chronic administration of GR in aged mice suppressed the age-related upregulation of pro-inflammatory genes (i.e., inducible nitric oxide synthase, cyclooxygenase-2, tumor necrosis factor-α, and interleukin-1β) in the hippocampus of aged mice, concomitant with improvements in cognitive function (Lee and Oh, 2015). Based on the notion of the Kampo formulation, these findings suggest that KKT can prevent age-related damage in organs, including the brain, through anti-oxidant and anti-inflammatory properties. In sum, the beneficial effects of KKT on behavior in aged mice may be orchestrated by the combination of possible neuromodulatory effects as discussed previously, and indirect effects on the whole-body including adaptability to oxidation and inflammation.

Our study had some limitations that should be noted. First, although we examined a variety of behaviors in young and aged mice, we lacked dose ranges of KKT and positive controls for each behavioral parameter in the IntelliCage assay. Second, we only used male mice in this study. Follow-up studies should address these limitations to elucidate the pharmacology of KKT in more detail, and validate the results presented here.

## Conclusion

Here, we demonstrated that KKT exerted diverse beneficial effects on CNS function in mice. KKT decreased anxiety and perseveration; accelerated decline in voluntary activity during the early part of the light phase; and enhanced consummatory hedonic responses, motivated behavior, and cognitive functions. Of note, the effects of KKT on diurnal rhythm, consummatory hedonic responses, and cognition were specific to aged mice. These findings suggest that KKT may normalize age-related functional decline and may be a potent therapeutic for elderly people with cognitive and mental frailty. Taking into consideration that the multifarious effects of KKT observed in this study cannot be explained by any single neurotransmitter system, and that KKT consists of numerous ingredients derived from as many as 14 medicinal herbs that are combined based on the Kampo concept, the mechanisms of its diverse effects are unlikely to be mediated *via* limited target molecules in the brain; rather, they more likely involve effects on multiple pathways. To identify the characteristics of each component in the KKT formula or to evaluate the influence of component combinations, further experiments that directly compare the efficacy of KKT with that of its components (e.g. GR and AR) are warranted. Furthermore, comparative assays between KKT and positive controls whose mechanisms of action are well characterized will help to elucidate the underlying mechanisms.

## Data Availability Statement

The raw data supporting the conclusions of this article will be made available by the authors, without undue reservation, to any qualified researcher.

## Ethics Statement

The animal study was reviewed and approved by The Experimental Animal Ethics Committees of Tsumura & Co.

## Author Contributions

HO, TE, YO, and KM contributed the conception and design of the study. HO and TE conducted the experiments. HO performed the statistical analyses. SM and MT validated the data. HO wrote the first draft of the manuscript. TE, YO and KM reviewed and edited the draft.

## Funding

This study was funded by Tsumura & Co and the funder was not involved in the study design, collection, analysis, interpretation of data, the writing of this article or the decision to submit it for publication.

## Conflict of Interest

All authors but TE are employees of Tsumura & Co. TE is a representative and a researcher of Phenovance Research & Technology, LLC. The authors declare that, except for income provided in the form of salaries from the employers, no financial support or compensation has been received from any individual or corporate entity and no conflict of interest exists.
