# Supplementary material for: Kamikihito Enhances Cognitive Functions and Reward-Related Behaviors of Aged C57BL/6J Mice in an Automated Behavioral Assay System
Source: Front Pharmacol. 2020 Jul 17;11:1037. doi: 10.3389/fphar.2020.01037 (PMC7379479; doi:10.3389/fphar.2020.01037)
Supplement: Supplementary file 1 [file DataSheet_1.docx]

***Supplementary Material***

| Name of Raw Material | Batch No. of Raw Material | |
| --- | --- | --- |
|  | KKT lot 351152900 | KKT lot 361095300 |
| *Astragali Radix* | 35105740 | 36100890 |
|  | 35102120 | 35103800 |
| *Bupleuri Radix* | 35105820 | 35105820 |
|  | 35104420 | 34102650 |
|  | 35103020 | 34102620 |
|  | 35104500 | 35103020 |
|  |  | 35104520 |
| *Ziziphi Semen* | 35107800 | 36104710 |
|  | 35106300 |  |
| *Atractylodis Lanceae Rhizoma* | 35105350 | 34103810 |
|  | 35103440 | 35105950 |
| *Ginseng Radix* | 35102220 | 35101270 |
|  | 35100880 |  |
| *Poria* | 35107930 | 36102810 |
| *Longan Arillus* | 34107790 | 35109510 |
| *Polygalae Radix* | 35107250 | 35107760 |
|  |  | 36101430 |
|  |  | 36100360 |
| *Gardeniae Fructus* | 35104170 | 36103940 |
|  | 35107130 | 36101460 |
|  | 35107280 | 36104610 |
| *Ziziphi Fructus* | 35106100 | 35108410 |
| *Angelicae Radix* | 35104060 | 36101580 |
|  | 35103740 | 35108640 |
|  | 35105220 |  |
| *Glycyrrhizae Radix* | 34102190 | 35101740 |
|  | 33109120 | 36100250 |
|  | 35104670 | 36101380 |
|  | 33105310 |  |
| *Zingiberis Rhizoma* | 35106270 | 36103460 |
| *Saussureae Radix* | 34104970 | 34104970 |

**Supplementary Table 1**. List of raw materials with batch number.


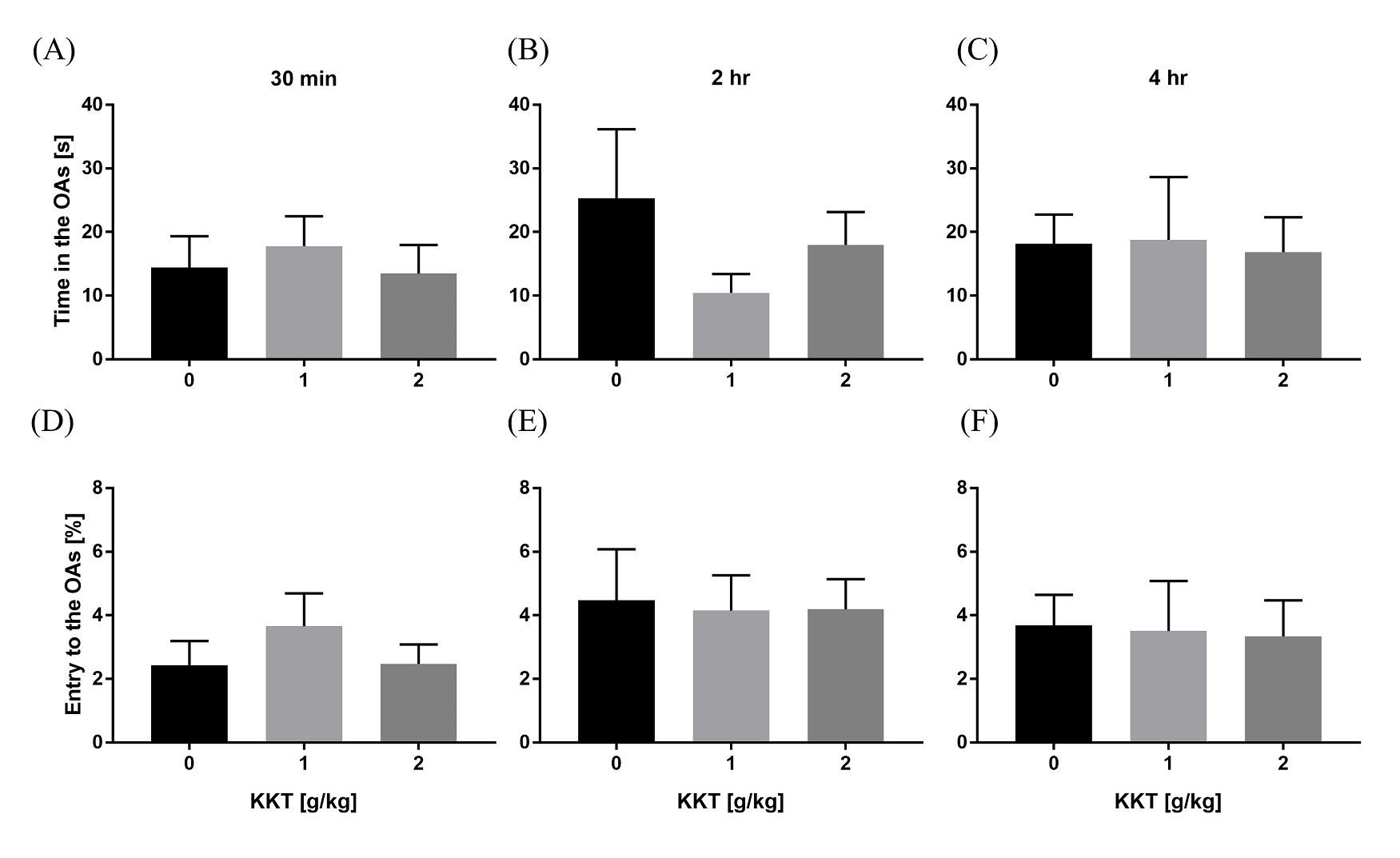


**Supplementary Figure 1**. Effect of single administration of KKT on exploratory behaviors in the EPM. **(A – C)** The time spent in the OAs measured 30 min **(A),** 2 hr **(B),** and 4 hr **(C)** after drug administration. **(D – F)** The percentage of OA entries measured 30 min **(D),** 2 hr **(E),** and 4 hr **(F)** after drug administration. Statistical significance was analyzed with Dunnett’s test. n = 15 – 16 **(A, D)** and 8 (**B, C, E, F).** All data are presented as the mean ± SEM.


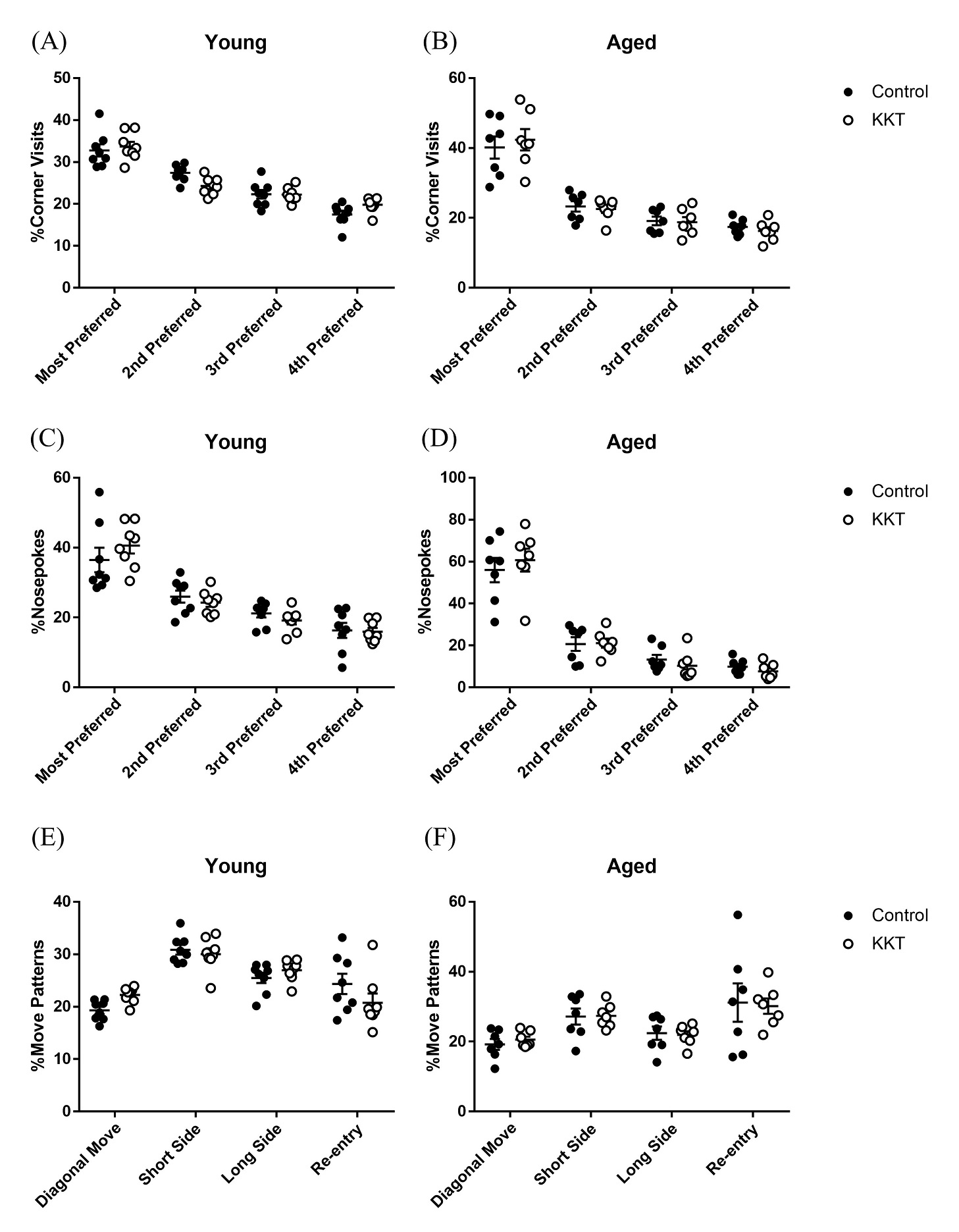


**Supplementary Figure 2**. Effect of KKT on basal movement patterns. **(A, B)** The percentage of each corner visits by young **(A)** and aged **(B)** mice. **(C, D)** The percentage of NPs performed at each corner by young **(C)** and aged **(D)** mice. **(E, F)** The percentage of movement patterns observed in young **(E)** and aged **(F)** mice. All data are expressed as scatterplots with the mean ± SEM; n = 8 (young) and 7 (aged). Statistical analysis was performed using Bonferroni’s multiple comparison.
